# Molecular Cloning, Carbohydrate Specificity and the Crystal Structure of Two *Sclerotium rolfsii* Lectin Variants

**DOI:** 10.3390/molecules200610848

**Published:** 2015-06-12

**Authors:** Vassiliki I. Peppa, Hemalatha Venkat, Anastassia L. Kantsadi, Shashikala R. Inamdar, Ganapati G. Bhat, Sachin Eligar, Anupama Shivanand, Vishwanath B. Chachadi, Gonchigar J. Satisha, Bale M. Swamy, Vassiliki T. Skamnaki, Spyridon E. Zographos, Demetres D. Leonidas

**Affiliations:** 1 Department of Biochemistry and Biotechnology, University of Thessaly, 26 Ploutonos Street, Larissa 41221, Greece; E-Mails: vikpeppa@gmail.com (V.I.P.), natassakan@windowslive.com (A.L.K.); vskamnaki@bio.uth.gr (V.T.S.); 2 Department of Biochemistry, Kuvempu University, Shimoga, Karnataka 577451, India; E-Mails: hemalatha.venkat@enzene.com (H.V.); satishlec@gmail.com (G.J.S.); 3 Department of Post Graduate Studies in Biochemistry, Karnatak University, Dharwad 580003, India; E-Mails: srinamdar2009@gmail.com (S.R.I.); ganapati.bd@gmail.com (G.G.B.); sachinbchem@gmail.com (S.E.); anupamas.biochem@gmail.com(A.S.); vishwanath1014@yahoo.co.in (V.B.C.); 4 Institute of Biology, Medicinal Chemistry & Biotechnology, National Hellenic Research Foundation, 48 Vas. Constantinou Ave, Athens 11635, Greece; E-Mail: sez@eie.gr

**Keywords:** *Sclerotium rolfsii* lectin, variant forms, gene constructs, glycan array, carbohydrate binding, X-ray crystallography

## Abstract

SRL is a cell wall associated developmental-stage specific lectin secreted by *Sclerotium rolfsii*, a soil-born pathogenic fungus. SRL displays specificity for TF antigen (Galβ1→3GalNAc-α-Ser//Thr) expressed in all cancer types and has tumour suppressing effects *in vivo*. Considering the immense potential of SRL in cancer research, we have generated two variant gene constructs of SRL and expressed in *E. coli* to refine the sugar specificity and solubility by altering the surface charge. SSR1 and SSR2 are two different recombinant variants of SRL, both of which recognize TF antigen but only SSR1 binds to Tn antigen (GalNAcα-Ser/Thr). The glycan array analysis of the variants demonstrated that SSR1 recognizes TF antigen and their derivative with high affinity similar to SRL but showed highest affinity towards the sialylated Tn antigen, unlike SRL. The carbohydrate binding property of SSR2 remains unaltered compared to SRL. The crystal structures of the two variants were determined in free form and in complex with *N*-acetylglucosamine at 1.7 Å and 1.6 Å resolution, respectively. Structural analysis highlighted the structural basis of the fine carbohydrate specificity of the two SRL variants and results are in agreement with glycan array analysis.

## 1. Introduction

Lectins are a group of monovalent or multivalent carbohydrate–binding proteins ubiquitously distributed in plants animals and fungi, that function as recognition molecules of specific glycan structures in cellular interactions [1]. Some lectins recognize tumor associated glycans and therefore have the potential to serve as biomarkers for malignant tumors and to assist in the study of changes in the glycosylation motif in cancer lines [2,3]. Furthermore, lectins modulate cancer associated signaling pathways emerging as new anti-cancer drugs [4]. The tumor-associated carbohydrate Thomsen-Friedenreich antigen (TF-Ag; Galβ1-3GalNAcα-*O*-Ser/Thr) is overexpressed on the cell surface in more than 90% of human cancer cells [5], contributing to cancer cell adhesion and metastasis [6]. Nowadays, TF antigen-binding lectins have attracted considerable interest since they have potential applications in cancer diagnostics and therapy. 

*Sclerotium rolfsii* lectin (SRL) is a cell wall-associated developmental–stage specific lectin secreted from the soil–born pathogenic fungus [7]. SRL is expressed on the mycelia at the time of sclerotial body formation and facilitates the aggregation of the mycelium to form sclerotial bodies by interacting with its endogenous glycosyl ceramide receptor(s), which have specific carbohydrate moieties [8]. SRL displays a clear specificity for TF and its substituted glycan structures [9]. During the last few years, our previous studies of SRL on various cancer cells demonstrated the population growth inhibitory effects on human colon and breast cancer cells, by inducing apoptosis *in vitro* and antitumor activity in HT29 xenografts *in vivo* [10]. SRL strongly inhibits the growth of human colon, ovarian and breast cancer lines, HT29, PA-1 and MCF-7, respectively [10,11,12] by the induction of apoptosis. SRL at subtoxic concentrations exerts tumour-suppressing effect *in vivo*. Furthermore, it exerts insecticidal activity on *Spodoptera litura* larvae by binding to membrane proteins of midgut epithelial cells and triggering caspase-3 dependent apoptosis [13]. The crystal structures of SRL both in the free form and in complex with *N*-acetyl galactosamine (GalNAc) and *N*-acetyl-d-glucosamine (GlcNAc) have been determined at 1.1 Å, 2.0 Å, and 1.7 Å resolution, respectively [7]. SRL-complex structures revealed that SRL has two distinct carbohydrate binding sites, a primary and a secondary one and elicited the structural determinants of the molecular recognition for carbohydrate binding. The carbohydrate specificity and promising ability of SRL to induce apoptosis in various human cancer cells prompted us to develop variant forms of SRL with altered physicochemical properties such as surface charge differences to increase the solubility and carbohydrate specificity to enhance the binding specificity of SRL against various forms of cancer. In the current study we report two recombinant variants SSR1 and SSR2, generated by the deliberate changes of the amino acids incorporated at particular sites of SRL by the synthetic gene construct method based on the complete amino acid sequence of SRL. Interestingly, SSR1 and SSR2 the two different variants of SRL, differ in their glycan specificity. In order to highlight the structural basis of the differences observed in sugar specificities, we have determined the crystal structure of the two variants, in their free form and in complex with GlcNAc. Glycan array analysis and structural studies have revealed that SSR1 binds to TF-antigen and GalNAc-α- (Tn antigen), whereas both SRL and SSR2 display specificity only for TF antigen. Therefore, SRL variants have the potential to be developed as therapeutic agents for cancer. 

## 2. Results and Discussion

### 2.1. Construction, Cloning and Sequence Determination of Sclerotium rolfsii Lectin Variants

Considering the significance of SRL to recognize cancer-associated TF antigen binding properties, full length synthetic genes (SSR1 and SSR2 ), which encode variant forms of SRL were chemically constructed based on the amino acid sequence data of the X-ray crystal structures and MALDI MS/MS analysis of SRL [7,14]. Since SRL had poor solubility and aggregated with loss of activity upon storage changes in amino acid sequence were introduced to enhance the solubility by altering the surface charge.

**Figure 1:**
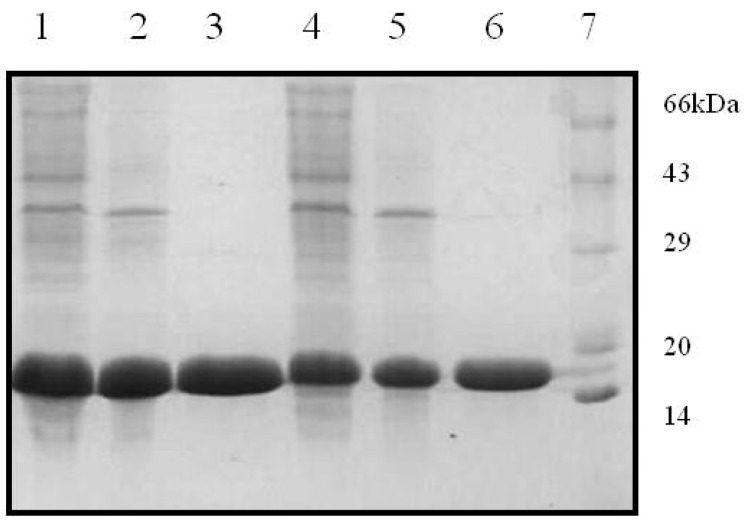
Purification profiles of SSR1 and SSR2 in 15% SDS-PAGE. Lane 1: SSR1 lysate, lane 2: DEAE flow through, Lane 3: Gel filtration elute, lane 4: SSR2 lysate, lane 5: DEAE flow through, lane 6: Gel filtration elute, and lane 7: Standard protein marker.

The two recombinant variants of SRL, SSR1 and SSR2 obtained encode for 141 and 143 amino acids, respectively. Restriction digestion analysis using NdeI and BamHI demonstrated that the two variants corresponded to gene lengths of 423 and 426 bps, respectively. The gene sequencing data confirmed the incorporation of the modified amino acids at the desired position in the primary sequence of SRL. Further the expression analysis of the recombinant proteins suggested that the lectins had monomeric molecular masses of ~16 kDa as determined by the SDS-PAGE analysis (Figure 1). In the current study attempts were made to incorporate the changes in the amino acid sequence of TF antigen binding lectin SRL were successful. The recombinant variants of SRL were expressed in a prokaryotic host, and purified to homogeneity, by the combination of ion exchange and gel filtration chromatographic methods. The hemagglutination assay of recombinant variants revealed that SSR1 and SSR2 agglutinated trypsinized human erythrocytes, irrespective of the blood groups, like SRL. The sequence based phylogenetic analysis has demonstrated that during the evolution, all the lectins derived from common ancestors and SRL and its variants maintains close relationship with ABL, a TF antigen binding lectin. Whereas other fungal lectin XCL which emerged much later than SRL during evolution also shares some similar properties with respect to structure as well as sugar binding ability. The common features of SRL and its variants with other fungal lectins observed with respect to their sugar binding ability and structural design are because of the conserved amino acids which contribute towards the specificity of the lectin.

### 2.2. Purification of SSR1 and SSR2 

The purification of the two SRL variants (SSR1 and SSR2) by ion exchange chromatography using DEAE-cellulose column has demonstrated that the recombinant lectins were eluted in unbound fractions when the column was washed with 25 mM TBS, pH 8.0 and remaining proteins were eluted using higher concentrations of NaCl. Recombinant variants partially purified by ion exchange chromatography were further purified by gel filtration chromatography. SDS-PAGE analysis showed that purified lectins are having monomeric mass corresponding to ~16 kDa. The purified recombinant variants, SSR1 and SSR2 agglutinated trypsinized human erythrocytes irrespective of the blood group.

### 2.3. Sequence Identity and Binding Site Analysis of SSR1 and SSR2

**Figure 2:**
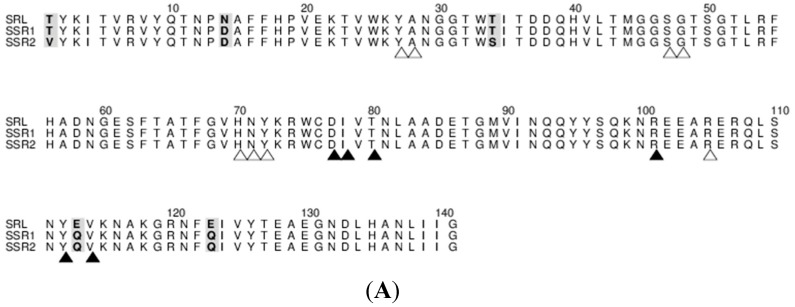

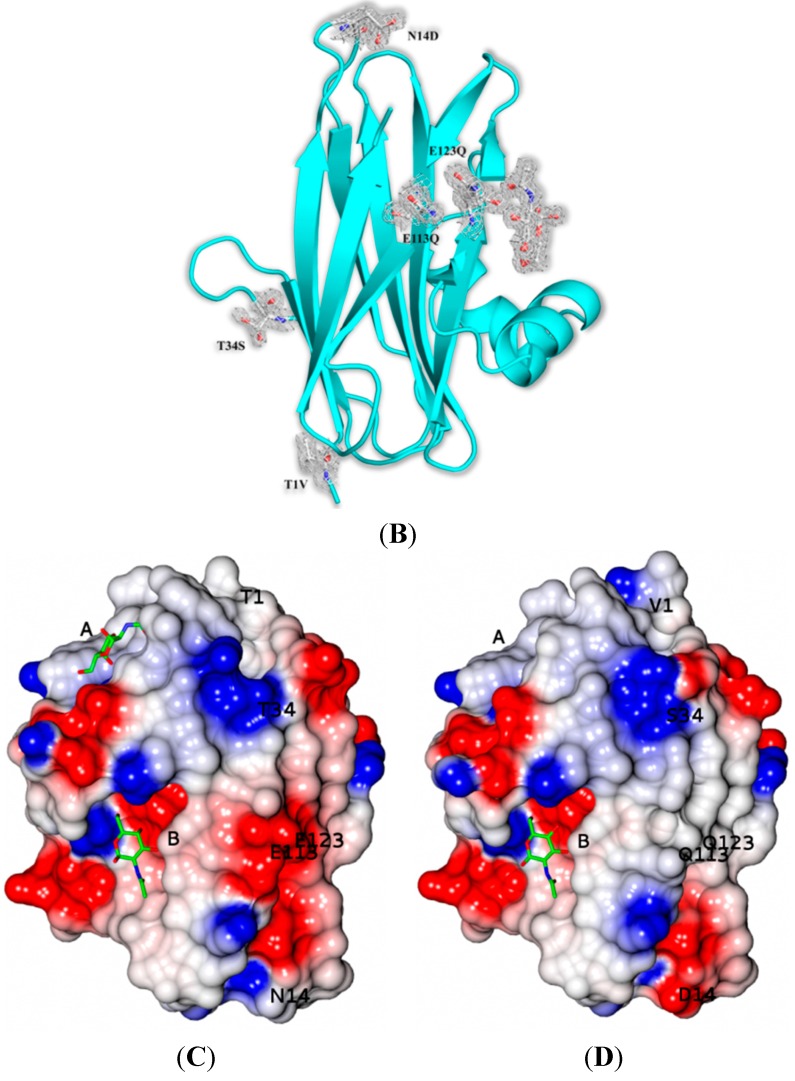
(**A**) Comparison of amino acid sequences of SSR1 and SSR2 with SRL. Mutated residues are in bold and shaded. Every 10th residue in the SRL sequence is numbered. Residues involved in carbohydrate ligand recognition are marked by open (primary) and closed (secondary) triangles; (**B**) A representation of the SSR2 structure with the mutated residues in their corresponding electron density calculated before incorporating the residues in the refinement. Electrostatic surface potential map of the SRL-GalNAc complex (**C**) and SSR2-GlcNAc complex (**D**). The carbohydrate molecules are shown as ball-and-stick models and the mutated residues are labelled. Electrostatic potential was calculated with the program CCP4MG [15] and is color coded on surface from blue (~5 kt/e) to red (~−5 kt/e).

The sequence analysis of the recombinant SRL variants demonstrated that there are no changes in the carbohydrate binding domain between SSR1 and SSR2 compared to SRL. Apart from the carbohydrate binding site residues other deliberate replacement of the amino acid is incorporated between SRL and SSR1 at 14th, 113th and 123rd positions, where the amino acids Asn, Glu and Glu residues were replaced by Asp, Gln and Gln, respectively. In case of SSR2 amino acids at the 1st, 14th, 34th, 113th and 123rd were replaced with Val, Asp, Ser, Gln and Gln, respectively (Figure 2).

### 2.4. Carbohydrate Binding Specificities of SSR1 and SSR2 as Determined by Glycan Array Analysis.

The glycan array analysis was carried out at the Consortium for Functional Glycomics (CFG), using mammalian printed array ver. 5.0 (Supplementary files S1 and S2 for SSR1 and SSR2, respectively) to determine the relative carbohydrate binding affinity of SSR1 and SSR2. The glycan array analysis has revealed that SSR1 recognizes Neu5Acα2-3GalNAcα-Sp8 (G#237), a sialylated-Tn antigen with highest affinity of RFU, 60561. It is interesting to note that these binding differences were observed only at the highest concentration of the protein used (200 μg/mL) and not at lower concentrations of SSR1 (20, 2 and 0.2 μg/mL). Additionally, SSR1 has also shown considerable affinity ranging from 36016 to 42247 RFU for GlcNAc/GalNAc linked to Tn (GalNAcα) antigen through α 1-3, β 1-3, or β 1-6 linkages (Table 2), signifying the importance of non-reducing GalNAcα moiety in SSR1’s specificity. The affinity of SSR1 towards TF (Galβ1-3GalNAcα) (βα) antigen is moderate compared to Tn with an affinity of RFU 37094 and slightly higher (39767) for β anomer (ββ) while it decreases significantly towards Galα1-3GalNAcα (αα) (Table 1). In contrast, SSR1 did not recognize Galα1-3GalNAcβ (αβ) (Table 3), showing its similarity with SRL in recognizing different anomers of TF disaccharide [9]. Although different techniques are essential to provide exclusive specificity, the relative binding affinity of SSR1 among different TF anomers can be assigned as TFββ > TFβα > TFαα >> TFαβ. The affinity of SSR1 drastically increases for TF disaccharide if C3 of galactose is substituted with sialic acid or GlcNAc or SO_4_ but decreases completely if the sugar is fucose (Supplementary file S1). Interestingly, SSR1 is also capable of recognizing N-linked glycans with high affinity (Supplementary file S3) which make this variant different from SRL which has been shown to recognize exclusively O-linked glycans [9]. 

**Table 1:** Relative binding of SSR1 to different glycans.

Glycan No.	Structure	RFU	SD
237	Neu5Acα2-3GalNAcα-Sp8	60561	2397
4	GalNAcα-Sp8	25418	2052
17	GlcNAcβ-Sp8	24386	857

**Table 2:** Binding affinity of SSR1 towards substituted form of Tn antigen.

Glycan No.	Structure	RFU	SD
193	GlcNAcβ1-6GalNAcα-Sp8	42247	886
180	GlcNAcβ1-3GalNAcα-Sp8	42113	1009
194	GlcNAcβ1-6GalNAcα-Sp14	41801	10768
95	GalNAcβ1-3GalNAcα-Sp8	40990	1513
181	GlcNAcβ1-3GalNAcα-Sp14	36016	6177

**Table 3:** Binding affinity of SSR1 towards different anomeric forms of TF disaccharides.

Glycan No.	Structure		RFU	SD
143	Galβ1-3GalNAcβ-Sp8	(ββ)	39767	1105
140	Galβ1-3GalNAcα-Sp8	(βα)	37094	1287
111	Galα1-3GalNAcα-Sp8	(αα)	17943	2523
113	Galα1-3GalNAcβ-Sp8	(αβ)	55	34

Another recombinant variant, SSR2 also recognizes the TF disaccharide and its derivatives with high affinity similar to SRL. Among all the O-glycans tested, SSR2 shows very high affinity towards sulphated TF disaccharide (G#29) with RFU of 60407 (Table 4), on the other hand it showed comparatively lower affinity (RFU of 38107) for unsubstituted TF disaccharide (Table 5). Strikingly, it has highest affinity for βα among all anomers of TF, making it more selective than native. Further, various substitutions such as SO_4_, Neu5Ac or GlcNAc at C3 of galactose in TF disaccharide significantly enhances the affinity of SSR2. It is important to note that though substitution of Neu5Ac or GlcNAc on galactose favors the binding of SSR2, affinity of these glycans decrease sharply if substitutions occurs on GalNAc of TF disaccharide (G#134, 135, 136 and 137 in Table 1). 

**Table 4:** Binding affinity of SSR1 towards TF disaccharide and its substituted forms.

Glycan No.	Structure	RFU	SD
29	(3S)Galβ1-3GalNAcα-Sp8	60407	578
243	Neu5Acα2-3Galβ1-3(6S)GalNAcα-Sp8	59832	1388
224	Neu5Acα2-3Galβ1-3GalNAcα-Sp8	56636	2693
89	GlcNAcβ1-3Galβ1-3GalNAcα-Sp8	55053	6128
167	Galβ1-4GlcNAcβ1-6(Galβ1-3)GalNAcα-Sp8	40694	1329
595	GlcNAcβ1-3Galβ1-4GlcNAcβ1-6(Galβ1-3)GalNAcα-Sp14	38968	2788
562	GlcNAcβ1-3Galβ1-4GlcNAcβ1-6(GlcNAcβ1-3Galβ1-3)GalNAcα-Sp14	38043	1942
134	GlcNAcβ1-6(Galβ1-3)GalNAcα-Sp8	37860	2198
567	GlcNAβ1-3Galβ1-3GalNAc-Sp14	32913	2103
600	Galβ1-4GlcNAcβ1-3Galβ1-3GalNAcα-Sp14	31899	1067
289	Neu5Acα2-3Galβ1-4GlcNAcβ1-6(Galβ1-3)GalNAcα-Sp14	30392	1803
135	GlcNAcβ1-6(Galβ1-3)GalNAcα-Sp14	29926	1115
168	Galβ1-4GlcNAcβ1-6(Galβ1-3)GalNAc-Sp14	29704	1107
568	Galβ1-3GlcNAcβ1-6(Galβ1-3)GalNAc-Sp14	29288	1294
603	Neu5Acα2-6Galβ1-4GlcNAcβ1-6(Galβ1-3)GalNAcα-Sp14	28023	3613
605	GlcNAcβ1-6(Neu5Acα2-3Galβ1-3)GalNAcα-Sp14	27706	943
345	GlcNAcα1-4Galβ1-3GalNAc-Sp14	27443	1913
360	KDNa2-3Galβ1-3GalNAcα-Sp14	25053	2863
601	Neu5Acα2-3Galβ1-4GlcNAcβ1-3Galβ1-4GlcNAcβ1-6(Galβ1-3)GalNAcα-Sp14	21093	1523
225	Neu5Acα2-3Galβ1-3GalNAcα-Sp14	20448	1701
591	Galβ1-4GlcNAcβ1-3Galβ1-4GlcNAcβ1-6(Galβ1-3)GalNAcα-Sp14	19429	826
244	Neu5Acα2-6(Neu5Acα2-3Galβ1-3)GalNAcα-Sp8	19265	1501
137	Neu5Acα2-6(Galβ1-3)GalNAcα-Sp14	13550	617
602	Neu5Acα2-6Galβ1-4GlcNAcβ1-3Galβ1-4GlcNAcβ1-6(Galβ1-3)GalNAcα-Sp14	9887	957
318	Neu5Acα2-3Galβ1-4GlcNAcβ1-6(Neu5Acα2-3Galβ1-3)GalNAcα-Sp14	9348	724
138	Neu5Acβ2-6(Galβ1-3)GalNAcα-Sp8	3150	1564
245	Neu5Acα2-6(Neu5Acα2-3Galβ1-3)GalNAcα-Sp14	3015	157
136	Neu5Acα2-6(Galβ1-3)GalNAcα-Sp8	374	199
63	Fuca1-2Galβ1-3GalNAcα-Sp14	287	95

In continuation of these results, fucosylated TF antigen is completely ineffective against SSR2 especially when it is attached to galactose through C2 carbon (Table 4) mimicking the binding property of SRL and SSR1 for this particular glycan.

Like SRL, this variant also showed higher affinity for βα anomer of TF (Galβ1-3GalNAcα) among all the anomers analyzed. This result is in sharp contrast to SSR1 which showed slightly higher affinity for β anomer (ββ) while SSR2 completely fails to recognize β anomer of TF disaccharide (Table 1). Furthermore, unlike SSR1, SSR2 doesn’t recognize any monosaccharides and Tn antigen as well as its substituted forms. This is an interesting and specific property of SSR2 which resembles SRL that also did not bind to any monosaccharides present in the array [9]. However, it is important to note that other glycan-lectin interaction studies such as surface plasmon resonance (SPR) [16] and isothermal calorimetry (ITC) [17] are essential to provide detailed subtle binding differences between SSR1 and SSR2. Apart from recognizing O-linked glycans, SSR2 also recognize many N-linked glycans present in the array (supplementary file S2) which is very similar property exhibited by both SSR1 and SSR2 unlike SRL.

**Table 5:** Binding affinity of SSR2 towards different anomeric forms of TF disaccharides.

Glycan No.	Structure	RFU	SD
140	Galβ1-3GalNAcα-Sp8	38017	1244
111	Galα1-3GalNAcα-Sp8	545	48
143	Galβ1-3GalNAcβ-Sp8	155	23
113	Galα1-3GalNAcβ-Sp8	30	14

### 2.5. Structural Studies 

The crystal structures of SSR1 and SSR2 lectins in their free form were determined at 1.7 Å and 1.6 Å resolution, respectively. Like SRL [7] there are two protein molecules (named mol A and mol B) in the crystallographic asymmetric unit of both variants related by a non-crystallographic two-fold symmetry. The two molecules pack antiparallel with their β-sheets inclined ~45° while the α-helices are located at the same side of the dimer. The mutations of SSR1 (N14D, E113Q, E123Q) and SSR2 (T1V, N14D, T34S, E113Q, E123Q) were all identified and all atoms of the mutated residues were well defined within the electron density map (Figure 2). A bound MPD molecule was found in both variants at the same place like in SRL [7] while in the SSR2 structure two additional molecules form the crystallization medium were found bound (MPD and a 2-amino-2-hydroxymethyl-propane-1,3 diol). Overall the crystal structures of SSR1 and SSR2 are similar to the SRL structure [7] and the introduction of the mutations did not seem to affect the overall structure of the protein. The rms displacement for all atoms between the SRL structure and those of SSR1 and SSR2 is 0.59 Å and 0.38 Å, respectively. In both structures of SSR1 and SSR2 the mutated residues, Asp14, Gln113, and Gln123 are located in a loop connecting strands S1 and F1, and in strands S4 and S5 respectively. In the structure of SSR2 the mutated residues Val1 and Ser34 are located in strands S1 and S2, respectively. The side chains of all mutated residues have the same conformation as their homologs in the SRL structure and do not induce any significant conformational change in their immediate environment. Only, the mutation N14D seems to cause a minor disturbance in the loop region of residues 12–14 in both variants (rms displacement of all atoms 0.18 Å and 0.26 Å from the SRL structure, respectively). In the SSR2 structure the T1V mutation leads to a shift of the residue by 0.6–1.0 Å (depending on the atoms) while the T34S mutation does not cause any conformational change on this residue. The surface potential of the two variants is reduced by ~10% both for the monomer and the dimers with respect to that of SRL. 

**Figure 3:**
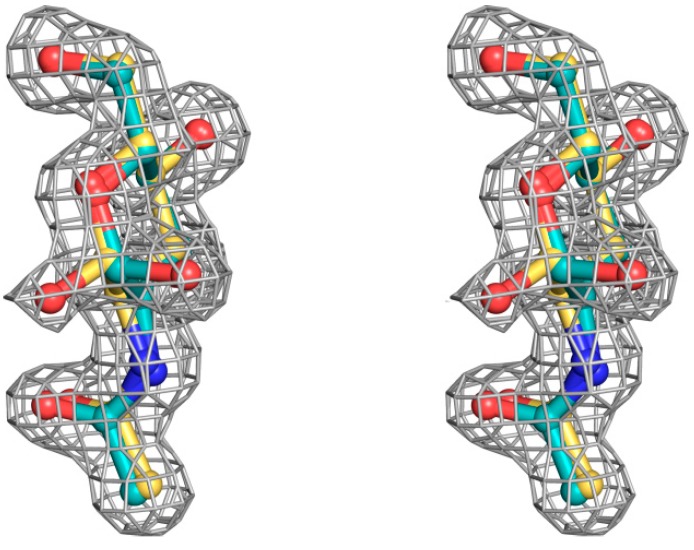
Stereoview of sigmaA 2|Fo|–|Fc| electron density map, contoured at the 1.0 σ level, for the *N*-acetyl β-d-glucosamine and *N*-acetyl α-d-glucosamine molecules bound at the secondary SSR1 carbohydrate-binding site before the incorporation of the carbohydrate ligand model.

In both the SSR1-GlcNAc and the SSR2-GlcNAc complexes, determined at 1.9 Å and 1.7 Å respectively, GlcNAc was found bound only in the secondary carbohydrate binding site of SRL in agreement with the complex of GlcNAc with SRL [7]. In the SSR1-GlcNAc structure an MPD molecule was found bound in the primary carbohydrate binding site similarly to the SRL-GlcNAc complex structure [7]. In the SSR2-GlcNAc complex the primary binding site is occupied by water molecules. All atoms of the ligand are well defined in both variant complexes, within the electron density map which has shown that GlcNAc binds to this site with two alternative conformations with respect to the hydroxyl group O1, α and β (Figure 3) that have equal occupancies. This finding is consistent with the ambiguity in the location of O1 hydroxyl group of both GlcNAc and GalNAc observed upon binding at the secondary binding site of SRL [7]. The binding of GlcNAc is almost identical in both molecules of the non-crystallographic dimer of both variant structures and thus we will only discuss the binding of this ligand in mol A. There are not any significant conformations upon binding of the ligand in each of the two variants. The rms displacement values for all atoms upon superposition of the SSR1-GlcNAc and the SSR2-GlcNAc structures onto the SRL-GlcNAc structure are 0.5 Å and 0.4 Å, respectively. 

**Figure 4:**
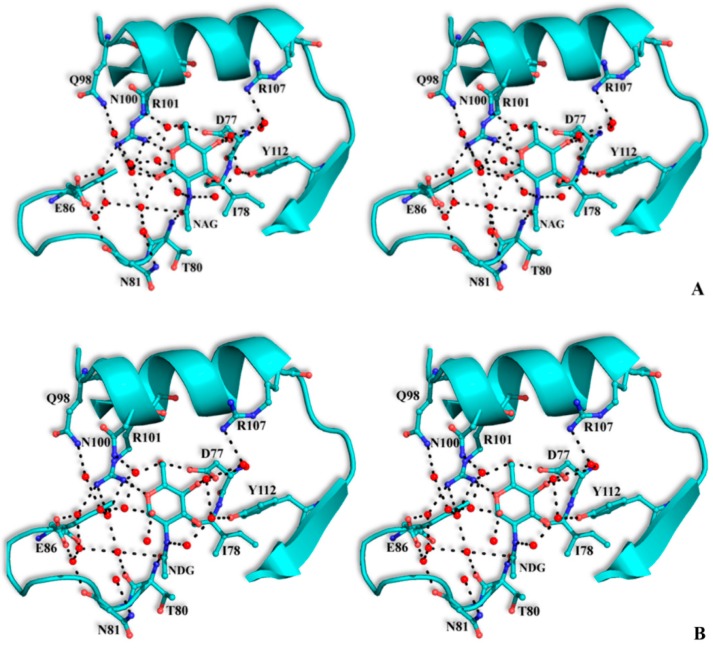

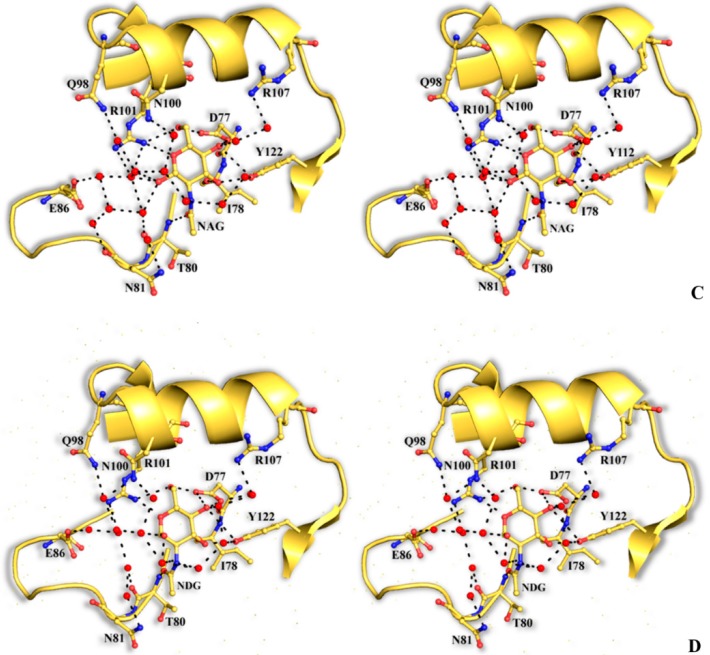
Stereoview of the carbohydrate interactions with the residues of SRL variants. SSR1 with *N*-acetyl β-d-glucosamine (**A**) and *N*-acetyl α-d-glucosamine (**B**); SSR2 with *N*-acetyl β-d-glucosamine (**C**) and *N*-acetyl α-d-glucosamine (**D**). The side-chains of protein residues are shown as ball-and-stick models. Bound water molecules are shown as blue spheres. Hydrogen bond interactions are represented by broken lines.

The structural mode of binding of GlcNAc does not differ significantly in the variants and the SRL structure. Briefly, GlcNAc is involved in hydrogen bond interactions with residues Asp77, Ile78, Thr80, Arg101, and Tyr112 (Table 6, Figure 4). All hydroxyl groups of GlcNAc except hydroxyl group O1, are involved in hydrogen bond interactions with protein residues, a fact that offers an explanation for the alternative conformations of this group. In both alternative conformations, the hydroxyl group O1, in the ligand complexes, is involved in hydrogen bond interactions only with water molecules. The existence of alternative conformations as well as the lack of protein interactions of hydroxyl group O1 is in agreement with the fact that this group is used to link GlcNAc with other sugar molecules in polysaccharides such as Neu5Acα2-3GalNAcα-Sp8 (G#237), a sialylated-Tn antigen with highest affinity of RFU (Table 1). GlcNAc atoms and 7 SRL residues participate in 19 (mol A) and 20 (mol B) van der Waals interactions at the secondary binding site.

**Table 6:** Potential hydrogen bonds of GlcNAc with SRL (data from [7]) and SRL variants in the crystal. In the SSR1 and SSR2 complexes the distance for the α and β conformations of GlcNAc is shown.

GlcNAc Atom	Protein Atom	Distance (Å)		
		SRL	SSR1	SSR2
O1	Water	2.8	3.2/2.9	2.7/2.8
O1	Water	2.8	2.6/2.7	3.2/2.7
O1	Water	-	3.3/-	3.1/-
O3	Ile78 O	2.7	2.6/2.6	2.6/2.6
O3	Tyr112 O^η^	2.6	2.8/2.6	2.7/2.6
O4	Asp77 O^δ1^	2.6	2.4/2.4	2.6/2.4
O4	Asp77 O^δ2^		3.3/3.3	-/3.3
O4	Tyr112 O^η^	3.3	3.2/3.2	3.3/3.3
O4	Water	2.6	2.7/2.7	2.5/2.7
O5	Arg101 N^η1^	2.9	3.0/3.0	3.0/3.0
O6	Asp77 O^δ2^	2.7	2.7/2.7	2.7/2.7
O6	Arg101 N^η1^	2.9	3.0/2.9	3.0/2.9
O7	Thr80 N	2.9	2.7/2.7	2.7/2.7
O7	Thr80 O^γ1^	2.9	3.2/3.0	3.2/2.9
O7	Water	-	3.1/3.3	3.2/-
N2	Water	2.9	2.9/3.0	2.8/2.9

Superposition of the ABL-T antigen (Galβ1–3GalNAc-α-*O*-Ser) complex structure (PDB entry: 1Y2X [18]) onto the SSR1 and SSR2 structures reveals that the GalNAc moiety of the T glycan structure can be accommodated in the SRR1 or SSR2 primary binding site without any steric conflicts by displacing six or seven water molecules, respectively. All SSR1 and SSR2 residues in this site adopt conformations very close to their structural equivalents in ABL favoring potential hydrogen bond interactions with the hydroxyl groups O2, O3, and O4 of the galactose moiety analogous to those observed in the ABL-T-antigen complex [18]. The serine moiety in SSR1, like in the ABL complex, could form van der Waals interaction with His70 and Arg105 but not with Tyr72 (the structural equivalent of Tyr74 in ABL). The shape complementarity between the surfaces of T-antigen and SSR1 and SSR2 in the primary binding site is 0.776 and 0.772 respectively (for SRL is 0.792 [7]). Therefore, all structural data show that both SSR1 and SSR2 can bind both the TF and the Tn antigen like SRL in disagreement with carbohydrate binding data.

**Figure 5:**
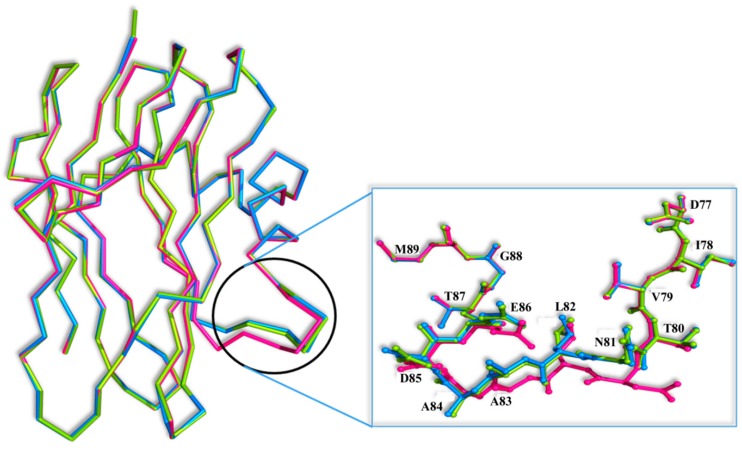
**S**uperposition of the Cα traces of the SRL (magenta), SSR1 (blue) and SSR2 (green) monomers. Inset: residues 81–86 are shown as ball-and-stick models.

Superposition of the SRL-GalNAc complex [7] onto the SSR1 and SSR2 structures reveals that GalNAc can easily bind at both the primary and secondary binding site by displacing five and three water molecules, respectively. However, there is a significant conformational change at the secondary binding site of the loop composed by residues 81–86 between the three structures (Figure 5). The most profound conformational difference is on SSR1 and especially on the side chain of Asn81 which moves away by 5 Å from its position in the SRL structure towards the secondary carbohydrate binding site. At this position the side chain atoms of Asn81 are not at a hydrogen bonding distance from the *N*-acetyl group of GalNAc. However, they are close enough (3.5 Å) to assume that upon binding they will make small shifts and they will be able to form hydrogen bond interactions with the ligand. The adoption of this conformation of Asn81 in SSR1 is made possible by a shift of the entire loop encompassing residues 81–86 by 3.0 Å on average, from their positions in the SRL structure. This shift is less profound in the SSR2 structure (1.0 Å on average) and does not lead to a change in the conformation of the side chain of Asn81 which maintains a similar conformation to that of the SRL structure. It is not clear whether this conformational change is the result of the mutated residues of SSR1. Examining the hydrogen bonding pattern of the mutated residues in the two variants it seems that mutations N14D, E113Q and E123Q (common in the two variants) do not alter the hydrogen bond and van der Waals interactions to neighboring protein residues. Similarly mutations T1V and T34S (SSR2) do not cause any change in the polar or non-polar interactions of these two residues in comparison to those observed in the SRL structure. 

## 3. Experimental Section 

### 3.1. Construction, Cloning and Sequence Determination of Sclerotium rolfsii Lectin Variants

The full length gene(s), which encodes *S.* *rolfsii* lectin (SRL), was chemically constructed with deliberate modifications based on the complete amino acid sequence determined by MALDI MS/MS analysis [14]. The modification of the lectin gene was carried out, in an intention to achieve the changes in physicochemical properties of the SRL. Further the gene(s) was released by digesting the plasmid (pUC57) using NdeI & BamHI (restriction enzymes), subsequently gene(s) was subcloned into pET20b, an expression vector, which was previously incised by the same restriction enzymes. Further the gene constructs were transformed into expression host BL21 DE3 (GOLD) strain of *E.* *coli*. Finally the clones were confirmed by restriction digestion and gene sequencing.

### 3.2. Expression Analysis of S. rolfsii Lectin Variants 

For the expression studies of SRL variant (SSR1 and SSR2), *E.* *coli* BL21 DE3 (GOLD) strain of *E.* *coli* harboring the SRL gene was precultured in 5.0 mL of Luria–Bertani medium containing 50 μg/mL ampicillin and incubated 37 °C for overnight with constant shaking (175 rpm). The overnight grown culture was subsequently inoculated into 1 L of the same medium and grown at 37 °C till the optical density at 600 nm reaches ~2.0. Further the cultures were induced using 250 μM isopropyl-β-d-thiogalactopyranoside (IPTG) and grown overnight at 18 °C with constant shaking. The overnight grown culture(s) was harvested by centrifugation at 4500 *g*, 4 °C for 10 min and the cell pellet (4 g) was suspended in 40 mL of 25 mM Tris-HCl pH 8.0 (Lysis buffer) containing 1 mM PMSF and 1 mM EDTA. The cell suspension was further subjected for lysis by sonication (60% amplitude for 20 min with pulse interval of 1 s). The cell suspension was centrifuged at 4500 *g*, 4 °C for 10 min and the supernatant containing lectin (crude extract) was collected. The lectin activity was determined by hemagglutination assay. The sample was subsequently, dialyzed against 25 mM Tris buffered saline (TBS/TB, pH 8.0) and used for purification. 

### 3.3. Purification of SSR1 and SSR2

The crude sample was dialysed against 25 mM Tris buffered saline (TBS, pH 8.0) loaded on to the DEAE ion exchange column (12 × 2 cm) which was previously equilibrated with equilibration buffer (TBS, pH 8.0). Flow through was collected as 3.0 mL fractions and column was subsequently washed with 25 mM TBS (pH 8.0). Lectin was eluted as unbound protein from the DEAE column. The fractions with hemagglutinating activity were pooled and dialysed against distilled water. After dialysis the sample was freeze-dried and the powder was suspended in 2.0 mL of 25 mM TBS, pH 7.5. Further, the sample was subjected to gel filtration chromatography column (Superdex G75) using an AKTA prime protein purification system. Fractions of 3 mL were collected and peak fractions with lectin activity were pooled and dialyzed against distilled water. The protein was quantified by the Lowry method [19] using bovine serum albumin as standard and sample was lyophilized and stored at −20 °C until further use. 

### 3.4. Hemagglutinating Activity and SDS-PAGE of SSR1 and SSR2

Hemagglutinating activity of the lectins were routinely determined as described previously [8]. Purified SRL variants were subjected to sodium dodecyl sulphate polyacrylamide gel electrophoresis in 15% (*w*/*v*) acrylamide gel according to Laemmli method [20] for determining homogeneity and the subunit molecular mass of the recombinant lectins. Subunit molecular mass (Mr) of SRL variants was estimated from the calibration curve obtained using standard protein markers.

### 3.5. Sequence Identity and Binding Site Analysis of SSR1 and SSR2

The deduced amino acid sequence of SSR1, SSR2 is compared with amino acid sequence of native *S.* *rolfsii* lectin using the Bioedit software for determining the differences in the binding sites of recombinant variants.

### 3.6. Glycan Microarray Screening for SSR1 and SSR2 

The carbohydrate binding specificity of SSR1 and SSR2 was determined by glycan microarray analysis using printed glycan array slides at the Consortium for Functional Glycomics (www.functionalglycomics.org). Micro array slides (printed array version 5.0) containing 611 naturally occurring and synthetic covalently attached glycans in replicates of six were used for the binding analysis as described by Blixt *et al.* [21]. In the binding assay, micro array slides were incubated with biotinylated lectins (200 μg/mL), in a binding buffer (20 mM Tris-HCl, pH 7.4 containing 150 mM NaCl, 2 mM CaCl_2_ and 2 mM MgCl_2_ containing 0.05% Tween 20 and 1% BSA) for 1 h at room temperature in a humidified chamber away from light. The slides were washed first with the same buffer without Tween 20 and then with water, and the bound lectin was detected using fluorescently labelled streptavidin. Fluorescent intensities of sample spots (RFU) for the 611 glycans were measured and analyzed using IMAGENE image analysis software (Bio Discovery, EI Segundo, CA, USA).

### 3.7. X-ray Crystallography

SSR1 and SSR2 crystals were grown using the hanging–drop vapor diffusion method by mixing equal volumes of a protein solution (16 mg/mL) in a 5mM Tris/HCl buffer, pH 8.5 and a solution of 20% *v*/*v* MPD. Tetragonal crystals (space group *P*4_2_2_1_2) were grown in 16 °C after 5 days. In these conditions both variants grow crystals that belong to space group *P*4_2_2_1_2 like SRL [22]. X-ray diffraction data were collected on an Oxford Diffraction SuperNova source diffractometer with a 135 mm Atlas CCD area detector using Nova microfocus Cu-K_α_ radiation source (λ = 1.54178 Å) at 100 K using a cryprotectant solution of the 25% *v*/*v* MPD. The GlcNAc complexes were formed by soaking SSR1 or SSR2 crystals at room temperature in a solution composed by 20 mM GlcNAc and 20% *v*/*v* MPD for 2 h prior data collection. Data processing was performed with CrysAlis^Pr^° [23] and scaled using SCALA from CCP4 program suite [24]. The crystal structures of the two variants were determined by the molecular replacement function of the program PHENIX [25] and the structure of SRL [7] as a search model. Building was performed with COOT [26], and refinement was performed using the maximum likelihood target function as implemented in PHENIX [25]. Ligand molecule coordinates and topologies were retrieved from the COOT ligand database and they were fitted to the electron density maps after adjustment of their torsion angles. Alternate cycles of manual rebuilding with the molecular graphic program COOT [24] and refinement with REFMAC [27] improved the quality of the models.

A summary of the data processing and refinement statistics is given in Table 7. The stereochemistry of the protein residues was validated by MolProbity [28]. Hydrogen bonds and van der Waals interactions were calculated with the program CONTACT as implemented in CCP4 [24] applying a distance cut off 3.3 Å and 4.0 Å, respectively. Figures were prepared with PyMol [29] and surface potential calculations with GRASP2 [30]. The coordinates of the new structures have been deposited with the RCSB Protein Data Bank (http://www.rcsb.org/pdb) with codes presented in Table 7.

**Table 7:** Summary of the diffraction data processing and refinement statistics. Values in parentheses are for the outermost cell.

Protein	SSR1	SSR2	SSR1–GlcNAc	SSR2–GlcNAc
Space group	*P*4_2_2_1_2	*P*4_2_2_1_2	*P*4_2_2_1_2	*P*4_2_2_1_2
Unit cell dimensions	a = b = 100.3 Å, c = 63.6 Å	a = b = 100.5 Å, c = 63.8	a = b = 99.9 Å, c = 63.8 Å	a = b = 100.0 Å, c = 63.8
Resolution (Å)	13.84–1.70	13.56–1.60	13.83–1.90	13.95–1.70
Outermost shell (Å)	1.79–1.70	1.69–1.60	2.00–1.90	1.79–1.70
Reflections measured	193,530 (16,850)	244,821 (22,780)	195,920 (17,118)	242,590 (21,336)
Unique reflections	36,246 (5329)	43,553 (4289)	25,956 (2553)	36,153 (5320)
Redundancy	5.4 (3.3)	5.6 (3.6)	7.5 (4.6)	6.7 (4.1)
R_symm_	0.044 (0.204)	0.080 (0.513)	0.089 (0.279)	0.097 (0.507)
Completeness (%)	99.4 (99.1)	100 (100)	99.9 (99.9)	99.7 (99.9)
	24.5 (5.5)	16.3 (2.2)	15.7 (5.2)	13.1 (2.3)
Wilson B factor (Å^2^)	9.53	9.89	8.56	13.25
R_cryst_	0.147 (0.161)	0.157 (0.217)	0.146 (0.181)	0.165 (0.259)
R_free_	0.179 (0.191)	0.188 (0.271)	0.186 (0.199)	0.203 (0.272)
No of solvent molecules	398	435	398	399
Deviation from ideality				
in bond lengths (Å)	0.014	0.014	0.010	0.010
in angles (°)	1.4	1.4	1.6	1.5
Average B factor				
Protein atoms (chain A/B)	8.4/6.3	11.9/9.3	8.5/6.2	11.8/9.9
Solvent molecules	19.4	24.3	20.7	24.0
Ligand atoms			7.4/13.5	7.6/13.2
PDB entry	4YLD	4Z2F	4Z2Q	4Z2S

## 4. Conclusions 

The cloning and the purification of two synthetic variant gene constructs of SRL are reported. SSR1 and SSR2 recognize TF antigen, but only SSR1 binds to Tn antigen. The glycan array analysis of the variants demonstrated that SSR1 recognizes TF antigen and its derivatives with high affinity like SRL but showed the highest affinity towards the sialylated Tn antigen, unlike SRL. The carbohydrate binding property of SSR2 remains unaltered compared to SRL. Their crystal structures both in the free form and in complex with GlcNAc have been determined at high resolution. Although a comparative structural analysis of the variant structures with the SRL (free and in complex with GlcNAc and GalNAc) structures show a significant conformational change of a loop (residues 81–86), and a 10% decrease in the surface potential, it does not reveal unambiguously the structural basis of their variance in carbohydrate specificity.

## Supplementary Materials

Supplementary materials can be accessed at: http://www.mdpi.com/1420-3049/20/06/10848/s1..
