# Supplementary material for: Molecular Cloning, Carbohydrate Specificity and the Crystal Structure of Two Sclerotium rolfsii Lectin Variants
Source: Molecules. 2015 Jun 12;20(6):10848–65. doi: 10.3390/molecules200610848 (PMC6272482; doi:10.3390/molecules200610848)
Supplement: Supplementary file 1 [file molecules-20-10848-s001.zip › Supplementary file S3.pdf]

## Supplementary Materials

**Table S3.** Relative binding affinity of N-linked glycan for SSR1.

| Glycan No. | Structure | RFU | SD |
|---|---|---|---|
| 452 | GalNAca1-3(Fuca1-2)Galb1-4GlcNAcb1-6(GalNAca1-3(Fuca1-2)Galb1-4GlcNAcb1-3)GalNAc-Sp14 | 53940 | 13202 |
| 581 | GlcNAcb1-3Galb1-4GlcNAcb1-3Galb1-4GlcNAcb1-3Galb1-4GlcNAcb1-3Galb1-4GlcNAcb1-2Mana1-6(GlcNAcb1-3Galb1-4GlcNAcb1-3Galb1-4GlcNAcb1-3Galb1-4GlcNAcb1-3Galb1-4GlcNAcb1-2Mana1-3)Manb1-4GlcNAcb1-4(Fuca1-6)GlcNAcb-Sp19 | 47884 | 3104 |
| 596 | GlcNAcb1-3Galb1-4GlcNAcb1-6(GlcNAcb1-3Galb1-4GlcNAcb1-3)GalNAca-Sp14 | 47868 | 11062 |
| 174 | GlcNAca1-3Galb1-4GlcNAcb-Sp8 | 46817 | 919 |
| 339 | GlcNAca1-4Galb1-4GlcNAcb1-3Galb1-4GlcNAcb1-3Galb1-4GlcNAcb-Sp0 | 46173 | 5139 |
| 547 | GlcNAcb1-3Galb1-4GlcNAcb1-3Galb1-4GlcNAcb1-2Mana1-6(GlcNAcb1-3Galb1-4GlcNAcb1-3Galb1-4GlcNAcb1-2Mana1-3)Manb1-4GlcNAcb1-4GlcNAcb-Sp25 | 45787 | 4329 |
| 579 | GlcNAcb1-3Galb1-4GlcNAcb1-3Galb1-4GlcNAcb1-3Galb1-4GlcNAcb1-2Mana1-6(GlcNAcb1-3Galb1-4GlcNAcb1-3Galb1-4GlcNAcb1-3Galb1-4GlcNAcb1-2Mana1-3)Manb1-4GlcNAcb1-4(Fuca1-6)GlcNAcb-Sp24 | 45417 | 1770 |
| 565 | GlcNAcb1-3Galb1-4GlcNAcb1-3Galb1-4GlcNAcb1-3Galb1-4GlcNAcb1-3Galb1-4GlcNAcb1-2Mana1-6(GlcNAcb1-3Galb1-4GlcNAcb1-3Galb1-4GlcNAcb1-3Galb1-4GlcNAcb1-3Galb1-4GlcNAcb1-2Mana1-3)Manb1-4GlcNAcb1-4GlcNAcb-Sp25 | 44919 | 1025 |
